# Development of a Wearable Torsion Sensor Utilizing a Geometric Model and a Hysteresis-Compensated Calibration Engine

**DOI:** 10.3390/s26206511

**Published:** 2026-10-14

**Authors:** Bonhak Koo, Seonghun Yu, Hojong Chang, Chansol Kang, Jeehyun Sim

**Affiliations:** 1 Digital Industry Division, Korea Dyeing & Finishing Technology Institute, Daegu 41706, Republic of Korea; rnqhsgkr29@dyetec.or.kr (B.K.);; 2 Department of National Public Policy, Graduate School, Chungnam National University, Daejeon 34134, Republic of Korea; ghwhd@cnu.ac.kr; 3 Department of Advanced Materials Engineering, Shinhan University, 95 Hoam-ro, Uijeongbu-si 11644, Republic of Korea; kangcs@shinhan.ac.kr

**Keywords:** algorithms, geometric modeling, motion tracking, strain sensors, torsion sensors

## Abstract

Measuring torsional movement is crucial for tracking human kinematics in applications such as virtual reality, biomechanics, rehabilitation, and soft robotic joints. However, continuous, non-invasive torsion measurement remains challenging due to multi-axial deformations, where existing methods rely heavily on empirical data fitting or computationally expensive deep learning models requiring vast training datasets. To address these limitations, this paper proposes a training-free, highly precise torsion estimation framework that converts soft resistive strain sensor data into forearm rotation angles using closed-form kinematic-geometric modeling. Two geometric representations—the Cylinder Model and Truncated Cone Model—are established to analytically map non-linear tensile strain to torsion angles. Additionally, an online calibration algorithm is integrated to mitigate cumulative measurement drift caused by viscoelastic hysteresis. Under static conditions, the proposed models achieved a high coefficient of determination (R^2^ = 0.9888) against reference IMU data. In dynamic trials, the hysteresis compensation algorithm maintained robust tracking performance (R^2^ = 0.8033), achieving an angular root-mean-square error (RMSE) of 4.12° and a cycle peak prominence relative error of 0.42% for the Truncated Cone Model. In this single-subject proof-of-concept feasibility study (*n* = 1), the proposed framework demonstrated the feasibility of continuous torsional motion tracking through a training-free geometric approach, providing a preliminary foundation for future evaluation in wearable systems and soft robotics.

## 1. Introduction

The demand for wearable motion capture and human–machine interfaces has grown rapidly in rehabilitation, athletic training, and physical therapy. For instance, quantitative monitoring of upper-limb movements, such as volleyball spiking, has been attempted using soft sensors attached to fingers and arms to overcome the line-of-sight occlusions associated with camera-based motion capture [1]. Similarly, wearable systems integrated with micro-Inertial Measurement Units (IMUs) have been deployed to analyze rotational swing paths in racket sports like tennis and golf [2]. In clinical rehabilitation, real-time upper-limb tracking using multi-axis inertial sensors and lightweight soft sleeves has shown immense promise for restoring motor functions in patients suffering from stroke or traumatic fractures [3]. Beyond passive tracking, wearable robotic devices and upper-limb exoskeletons have garnered increasing attention for their significant clinical and occupational usefulness. In industrial and occupational ergonomics, repetitive upper-limb tasks and heavy physical workloads frequently lead to work-related musculoskeletal disorders (WMSDs), particularly in the shoulder, back, and forearm. Recent investigations employing muscle network analysis have demonstrated that active and passive occupational exoskeletons can substantially alleviate muscular fatigue, redistribute biomechanical loads, and preserve coordinated neuromuscular patterns during dynamic or bilateral overhead lifting tasks [4]. Concurrently, in neuro-rehabilitation scenarios (e.g., chronic stroke survivors and patients with Parkinson’s disease), robotic exoskeletons and assisted movement therapies play a critical role in promoting neuroplasticity, restoring impaired muscle synergies, and re-establishing functional inter-muscular coordination [5,6,7,8]. To maximize these therapeutic and preventive benefits, soft and compliant wearable sensing technologies—capable of continuously tracking multi-degree-of-freedom joint rotations (e.g., forearm pronation/supination) without hindering natural kinematics—are indispensable for providing precise feedback control and ergonomic biofeedback.

Similarly, these multi-axial motion tracking challenges extend to the robotics domain, where compliant proprioceptive feedback is a fundamental requirement in modern robotics, especially in soft robotics and smart actuation systems. While traditional rigid exoskeletons and robotic devices offer substantial mechanical assistance, their rigid links often impose kinematic constraints and unnatural loading on the human body. In this context, soft robots not only overcome the heaviness and bulkiness of rigid exoskeletons, but also ensure fewer restrictions on joint range of motion and higher transparency with the neuromuscular system [9,10]. However, traditional rigid robots and exoskeletons rely on heavy, bulky rotary encoders to measure joint angles, which are incompatible with such compliant and lightweight structures. To achieve high dexterity while preserving biomechanical transparency, soft robotic joints, wearable assistive suits, and artificial muscles—such as dielectric elastomer actuators (DEAs), shape memory alloys (SMAs), and pneumatic artificial muscles (PAMs)—urgently require highly integrated, flexible, and responsive feedback sensors to track multi-axial deformation, particularly torsion, in real-time. To address these kinematic feedback requirements, several conventional and emerging sensing modalities have been investigated, each presenting distinct trade-offs in wearability, signal fidelity, and mechanical integration:

1. ELECTROMYOGRAPHY (EMG) & TORQUE SENSING

Electromyography measures muscle excitation levels during physical activities [11]. Beyond simple motion or workout classification [12], EMG has been widely employed as a vital evaluation paradigm to assess neuromuscular load, muscle network coherence, and muscular synergy reorganization during interaction with active and passive occupational exoskeletons [4,5,6,7,8,9]. However, surface EMG requires precise skin preparation, is highly susceptible to motion artifacts, causes skin irritation during long-term wear, and cannot directly map continuous multi-axial joint kinematics (such as pure forearm torsion) without highly complex musculoskeletal modeling and individualized anatomical parameterization. To overcome the anatomical modeling dependencies and bio-potential acquisition difficulties of EMG, kinematic-based tracking using Inertial Measurement Units (IMUs) has emerged as an alternative approach.

2. INERTIAL MEASUREMENT UNITS (IMU)

IMUs fuse data from accelerometers, gyroscopes, and magnetometers to determine orientation trajectories [13]. By providing wearable and autonomous orientation tracking, IMUs effectively overcome the intrinsic limitations of conventional optical camera-based motion capture systems, such as vulnerability to ambient illumination, lack of portability outside laboratory environments, and line-of-sight occlusion [14]. Nonetheless, IMUs are prone to integration drifts over time, suffer from magnetic field interference, and inherently struggle to decouple pure torsional twist from concomitant bending on soft bodies. In response to the rigidity, drifting vulnerabilities, and mechanical constraints of IMUs, soft and flexible strain sensors have garnered intensive attention as conformable sensing skins for continuous kinematic tracking.

3. FLEXIBLE RESISTIVE STRAIN SENSORS

Elastomeric and textile-based strain sensors convert mechanical stretching into electrical resistance signals [15]. In addition to resistive mechanisms, flexible capacitive and piezoelectric sensors have also been actively investigated for human motion monitoring, offering advantages in baseline stability and dynamic responsiveness, respectively. Due to their high mechanical compliance and seamless integration, these soft sensors have been widely applied in health monitoring [16,17,18], body motion tracking [19,20], and soft robotic proprioception [21]. However, while these soft sensors conform well to body kinematics, establishing a reliable, deterministic transfer function without extensive empirical fitting remains unaddressed for multi-axial torsion.

Despite their potential, establishing a reliable relationship between soft sensor readings and high-degree-of-freedom motions like torsion remains a bottleneck [22,23,24,25]. Prior torsion-sensing literature predominantly relies on existing empirical-fitting-based methods, which lack structural causality and fail when the sensor is transferred to a user with a different arm geometry or a robot joint with a different aspect ratio. To situate the novelty of this work against these limitations, this study introduces a closed-form geometric approach. To achieve precise, real-time closed-loop control in smart robotic systems and wearable sleeves, this mathematically rigorous yet computationally simple kinematic modeling is required.

In this study, a soft wearable sensing system is proposed that directly translates the electrical resistance variations of a sleeve-mounted strain sensor into continuous forearm pronation/supination angles without relying on empirical training datasets. To achieve this, two distinct analytical geometric models are formulated (Cylinder and Truncated Cone) based on the structural boundaries of the rotating link. Furthermore, to overcome the inherent viscoelastic hysteresis of soft elastomers, an online baseline-drift compensation algorithm is implemented. By effectively minimizing dynamic tracking errors and baseline drift, this framework provides dependable kinematic states essential for closed-loop adaptive control—ensuring phase-synchronized robotic assistance without mechanical resistance—as well as reliable biofeedback integration for continuous rehabilitation and ergonomic monitoring. The preliminary technical feasibility and measurement performance of the proposed framework are evaluated in a single-subject proof-of-concept feasibility study (n = 1) against multi-axis IMU data under both static and dynamic movement conditions.

The remainder of this paper is structured as follows. Section 2 details the characteristics of the soft strain sensor, the analytical derivation of the two kinematic-geometric models (Cylinder and Truncated Cone), the hysteresis-drift compensation algorithm, and the experimental protocol. Section 3 presents the static and dynamic experimental results and comparative accuracy evaluations. Section 4 discusses the physical and functional implications of the findings, including the significance for closed-loop control, as well as the current limitations of the system. Finally, Section 5 concludes the paper and outlines prospective avenues for future work in soft robotic applications. 

## 2. Materials and Methods

### 2.1. Torsion Sensor Assembly and Hardware Integration

To capture torsional forearm rotation, the system utilizes a lightweight, wearable sensing assembly consisting of an elastic fabric sleeve, a single soft resistive strain sensor, and an embedded signal acquisition circuit. Measurement of torsion in the human body is essential for kinematic tracking in applications such as health monitoring, virtual reality (VR), neuro-rehabilitation, and soft robotic joint proprioception [26,27]. However, forearm pronation and supination involve non-linear three-axial deformation around the longitudinal radius–ulna axis rather than simple planar flexural bending [28,29,30], presenting unique challenges for continuous stretch-to-angle transduction without empirical overfitting [31]. 

To address these sensing constraints, a dedicated wearable torsion sensing sleeve was constructed. The underlying substrate consists of a custom-tailored compression forearm sleeve fabricated from a dual-blend elastic textile composed of 85% nylon and 15% spandex. This breathable, high-recovery textile provides conforming skin adherence without excessive contact pressure, effectively minimizing migration and interface slipping during repeated cyclic pronation and supination. For real-time signal acquisition, the integrated strain sensor was interfaced with a dedicated voltage divider circuit incorporating a fixed carbon film reference resistor (*R_ref_* = 1 kΩ, ±5%). The analog potential across the strain sensor was sampled using the 10-bit analog-to-digital converter (ADC) of an Arduino Uno microcontroller board (Arduino S.r.l., Monza, Italy) operating at a stable sampling frequency of 10 Hz. The digitized voltage signals were converted to instantaneous resistance values and streamed in real-time to a host computer for downstream digital filtering and geometric angle estimation.

Additionally, capturing torsional motion around a slender anatomical link requires careful sensor placement and kinematic consideration due to the relatively small rotational radius compared to simple hinge joints. Prior investigations on torsion sensing predominantly relied on direct empirical correlations between raw resistance changes and applied twist angles without structural kinematic modeling [26,27,32], which inherently lacks physical causality and limits sensor transferability across different morphological dimensions [33]. Furthermore, previous attempts using flexible piezoelectric wristbands encountered severe measurement constraints due to the intrinsic bending stiffness of the piezoelectric substrate and restricted gauge lengths [34], and were also limited to assessing angular velocity ranges (25–40°/s) rather than tracking continuous absolute rotation angles.

**Figure 1:**
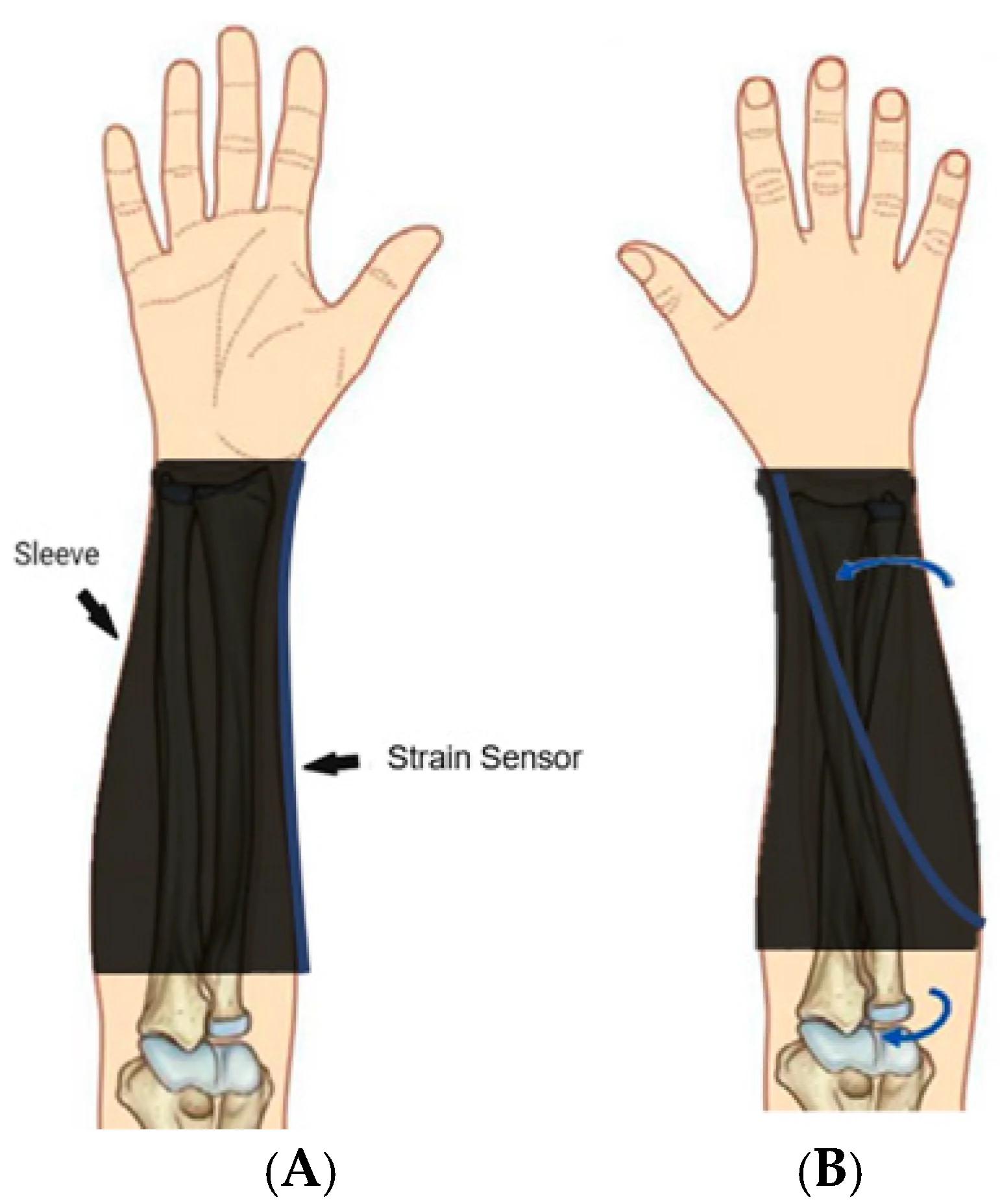
Torsion position. (**A**) Palm anterior, and (**B**) palm posterior.

To resolve the limitations of conventional planar wristbands and achieve continuous angular tracking, the single elastomeric cord sensor in this study was strategically anchored along a diagonal helical trajectory on the compression sleeve (Figure 1). Anatomically, forearm pronation and supination occur as the radius rotates around the ulna along a longitudinal axis extending from the elbow joint to the distal radio-ulnar center. When applied to compliant soft actuators, these movements correspond directly to clockwise and counter-clockwise torsional rotations. By accommodating these rotations within the high elastic limit of the silicone-carbon cord, this assembly successfully transduces continuous multi-axial twist into measurable axial elongation, providing the physical foundation for the analytical geometric models established herein.

### 2.2. Sensor Properties, Sleeve Integration, and Attachment Location

To capture forearm torsion, a commercial conductive rubber cord stretch sensor (Adafruit Industries, New York, NY, USA; distributed by Device Mart (NTREX Co., Ltd., Incheon, Republic of Korea)) was integrated into an elastic wearable sleeve. The sensor consists of an elastic silicone elastomer matrix impregnated with conductive carbon black particles, featuring an outer diameter of 2.0 mm and an active gauge length of *L*_0_ = 70 mm. In an unstretched resting state, the sensor exhibits a nominal linear resistance of 140–150 Ω/cm, corresponding to an initial baseline resistance (*R*_0_) of approximately 1 kΩ. As the cord sensor elongates, the conductive carbon pathways within the silicone matrix separate, yielding a monotonic increase in electrical resistance. The sensor is anchored onto the compliant sleeve fabric along the designated anatomical trajectory using flexible textile stitching at both terminal ends, allowing free axial stretch during movement without restricting the compliance of the sleeve or detaching at the interface.

**Figure 2:**
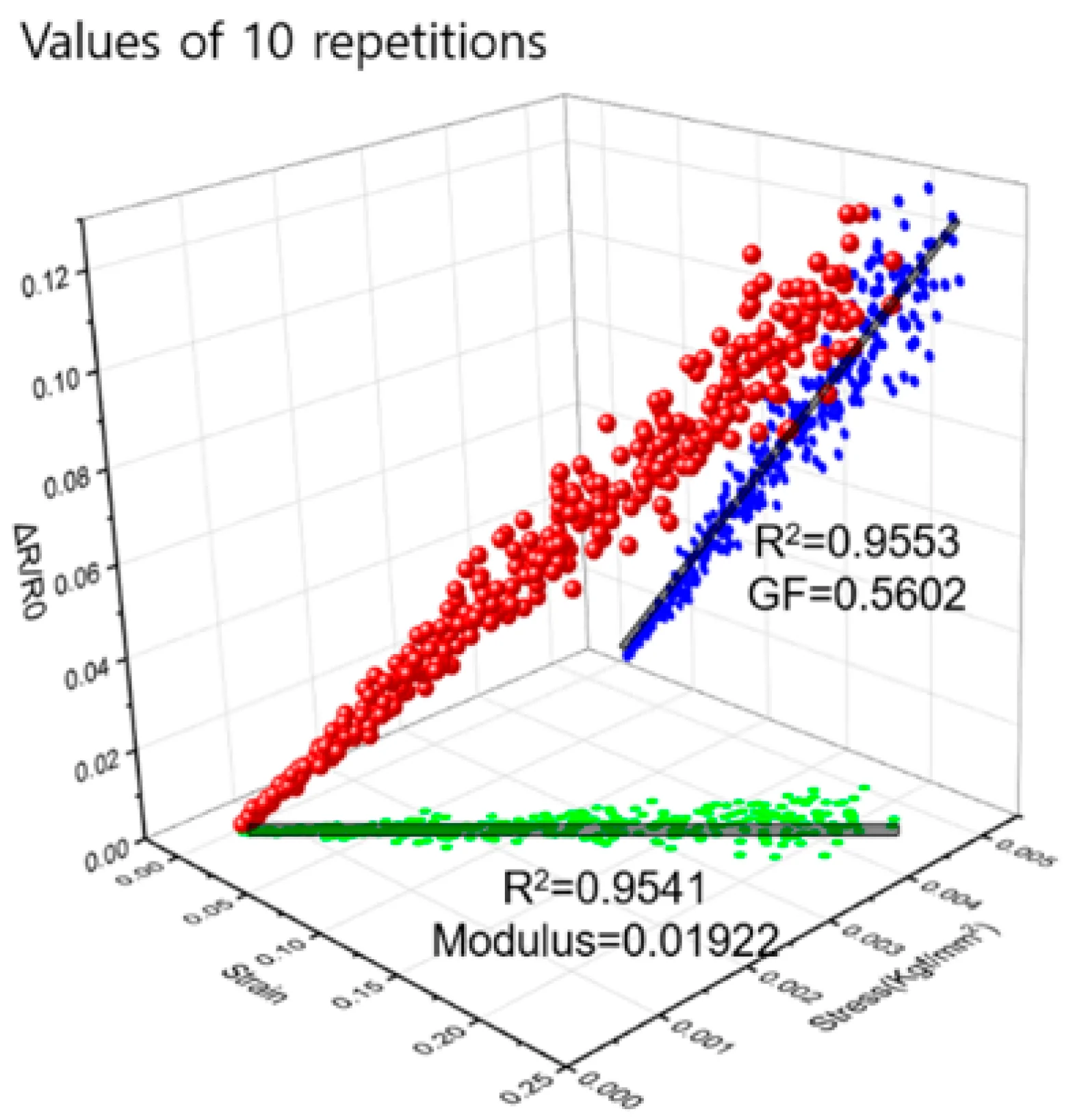
The modulus and gauge factor of the sensor. Blue and green data points correspond to the gauge factor (GF = 0.5602, R^2^ = 0.9553) and modulus (0.01922, R^2^ = 0.9541), respectively. Red data points are included for visualization and do not represent a separate material parameter.

Through the use of a Universal Testing Machine (UTM, FGA-5, DACELL), the strain-stress-resistive change was graphed and fitted with a linear regression. As characterized in Figure 2, the 70 mm conductive rubber cord sensor was slowly subjected to tension at a speed of 1 mm/s within a strain range of 0.25. The modulus was measured as 0.01922 with a coefficient of determination (R-squared) of 0.9541. The available strain range suggests that the sensor has sufficient mechanical extensibility for forearm torsion measurements within the range of anthropometric dimensions and pronation/supination torque values reported in the literature [35,36]. Since modulus exhibited linearity with a coefficient of determination of 0.9873 up to a strain of 1.21 in one cycle, it is determined that the tension changes occur within the elastic limit, making it highly suitable for elastic mechanical and biological systems. To quantitatively evaluate the electromechanical performance of the sensor, key parameters including sensitivity, recoverability, and hysteresis were assessed across the 10 repeated cyclic loading–unloading trials within a strain range of 0.25. The gauge factor (sensitivity) was measured as 0.5602 with a coefficient of determination of 0.9553. Given the relatively low gauge factor of less than 1, it was deemed suitable for measurements as it can accommodate the substantial movements of both human joints and soft actuators [37]. Upon release of applied tensile strain, the sensor demonstrated an elastic recoverability of 96.8% toward its baseline state. The degree of hysteresis—quantified as the maximum resistance discrepancy between the loading and unloading curves normalized by the dynamic resistance span—was evaluated to be 11.4%. Although this intrinsic viscoelastic hysteresis caused an intra-trial baseline elevation, its bounded repeatability enabled the online calibration engine (Algorithm B) to effectively compensate for drift without confounding actual kinematics.

**Figure 3:**
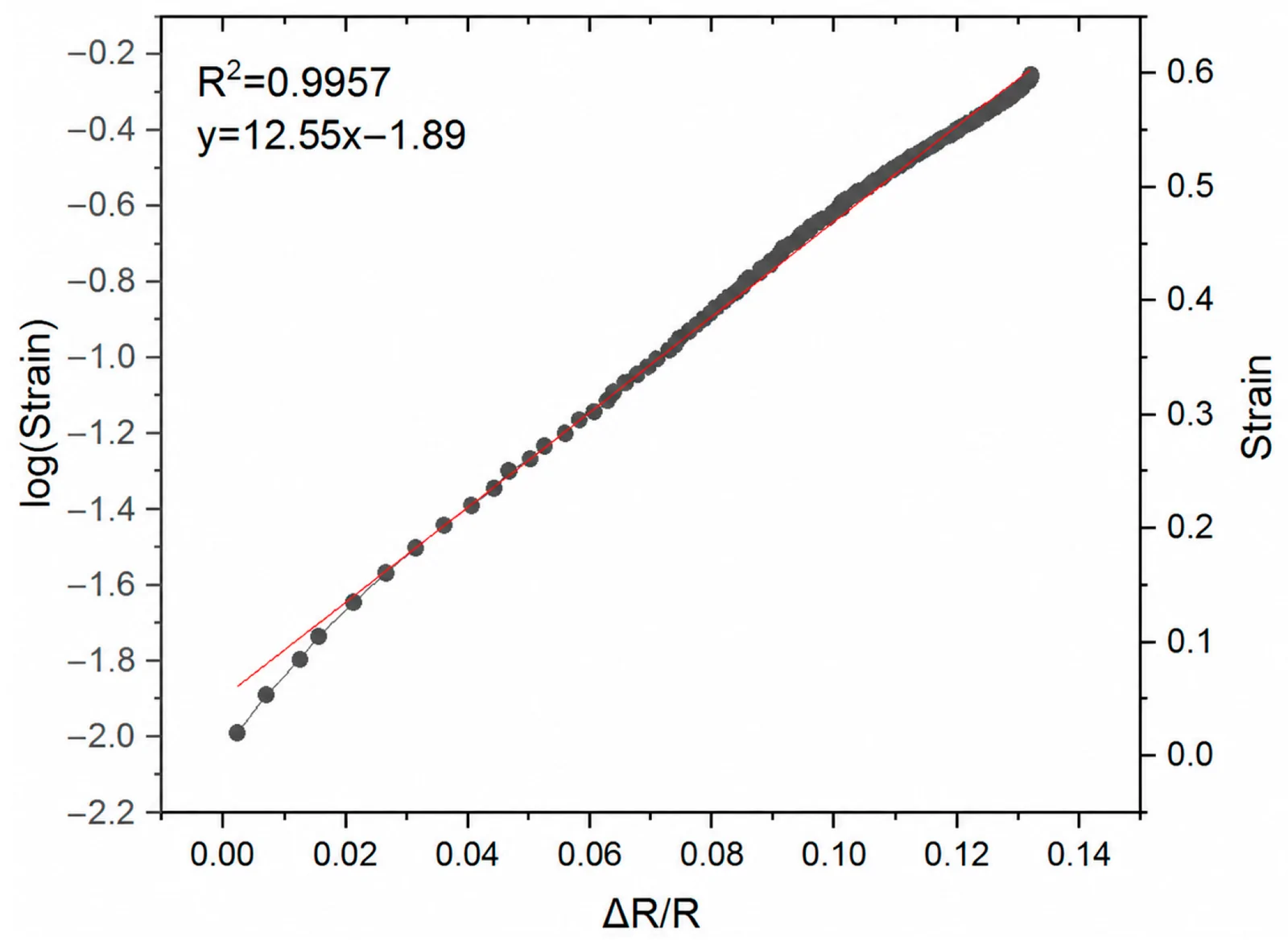
The relationship between resistance change rate and tensile strain.

The resistance of the strain sensor can be converted into strain using the relationship between the resistance rate and log(strain). As shown in Figure 3, a linear relationship was established using a semi-log equation to estimate strain based on resistance change. The coefficient of determination (R-squared) was 0.9957, indicating that strain can be calculated from the resistance rate as a linear relationship. The reason for using the resistance rate instead of pure resistance values is that the resistance rate is an intrinsic property of the sensor resistance, independent of the initial length setting. The resistance values obtained through Arduino were based on setting the initial resistance value and then obtaining the resistance change, from which the resistance rate was calculated. Strain is the rate of change in sensor length, calculated by setting the initial length value and then obtaining the length change. The equations are as follows: 

*R*_0_: Initial resistance, *R*: Final resistance, *L*_0_: Initial length, *L*: Final length.

(1) $$\frac{\left(R-R_{0}\right)}{R_{0}}=\frac{\Delta R}{R_{0}}$$

(2) $$\frac{\left(L-L_{0}\right)}{L_{0}}=\varepsilon$$

To prevent methodological confusion, it is essential to distinguish between the intrinsic material characterization of the raw elastomeric sensor and the kinematic motion mapping. The semi-logarithmic regression described above is strictly an offline, one-time calibration to establish the intrinsic electro-mechanical property (i.e., the constant gauge factor) of the conductive rubber material, which remains user-independent. In contrast, the “training-free” claim of this framework strictly designates the kinematic angle estimation phase. Unlike conventional learning-based or empirical mapping approaches that require extensive, subject-specific motion datasets to fit non-linear rotational trajectories, the proposed framework estimates continuous torsion angles purely through closed-form geometric formulations (the Cylinder and Truncated Cone Models). Once basic physical anthropometric parameters (*r*, *R*, *L*_0_) are configured, the framework analytically maps elongation to torsion without collecting prior user movement datasets or executing data-driven algorithm training.

**Figure 4:**
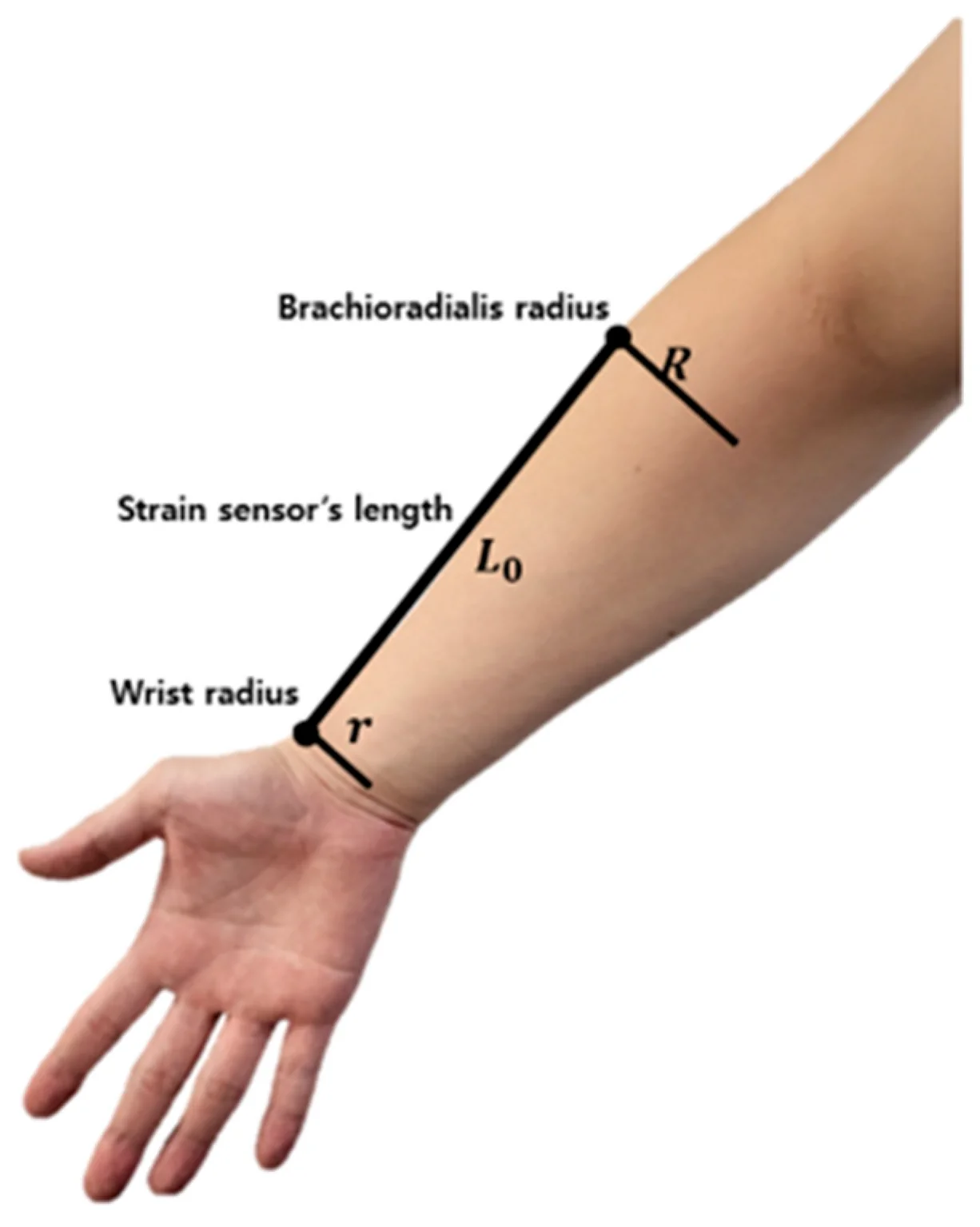
Sensor attachment location and measurement areas for modeling.

The sensor was anchored along the superficial dorsal-ulnar boundary of the forearm, spanning from the proximal belly of the brachioradialis muscle to the distal wrist (Figure 4). This longitudinal span was selected because the proximal forearm experiences minimal rotational displacement relative to the elbow joint, whereas the distal wrist undergoes the maximum angular excursion during pronation and supination, providing the greatest kinematic sensitivity. Crucially, routing the sensor along the subcutaneous border of the ulna effectively leverages an anatomical region with minimal underlying muscle bulk. Consequently, this trajectory decouples the sensor from the prominent radial expansion induced by activation of the brachioradialis and deep forearm flexors/extensors during rotation, ensuring that measured resistance variations are predominantly governed by true torsional elongations rather than volumetric muscle deformation. To parameterize the analytical geometric models, three physical measurements were acquired: the distal wrist radius (*r*), the proximal radius at the brachioradialis landmark (*R*), and the initial distance between the two anchoring points (*L*_0_).

### 2.3. Geometric Modeling

To establish structural causality between sensor elongation and rotational kinematics, the forearm torsion mechanics were formulated using two analytical representations: the Cylinder Model (CM) and the Truncated Cone Model (TCM). All models assumed rotation around a longitudinal axis extending from the elbow to the center of the wrist. The initial baseline posture was defined as the palm-anterior position (0° in Figure 1A), whereas the maximum pronation was set to the palm-posterior position (180° in Figure 1B).

#### 2.3.1. Cylinder Model (CM)

**Figure 5:**
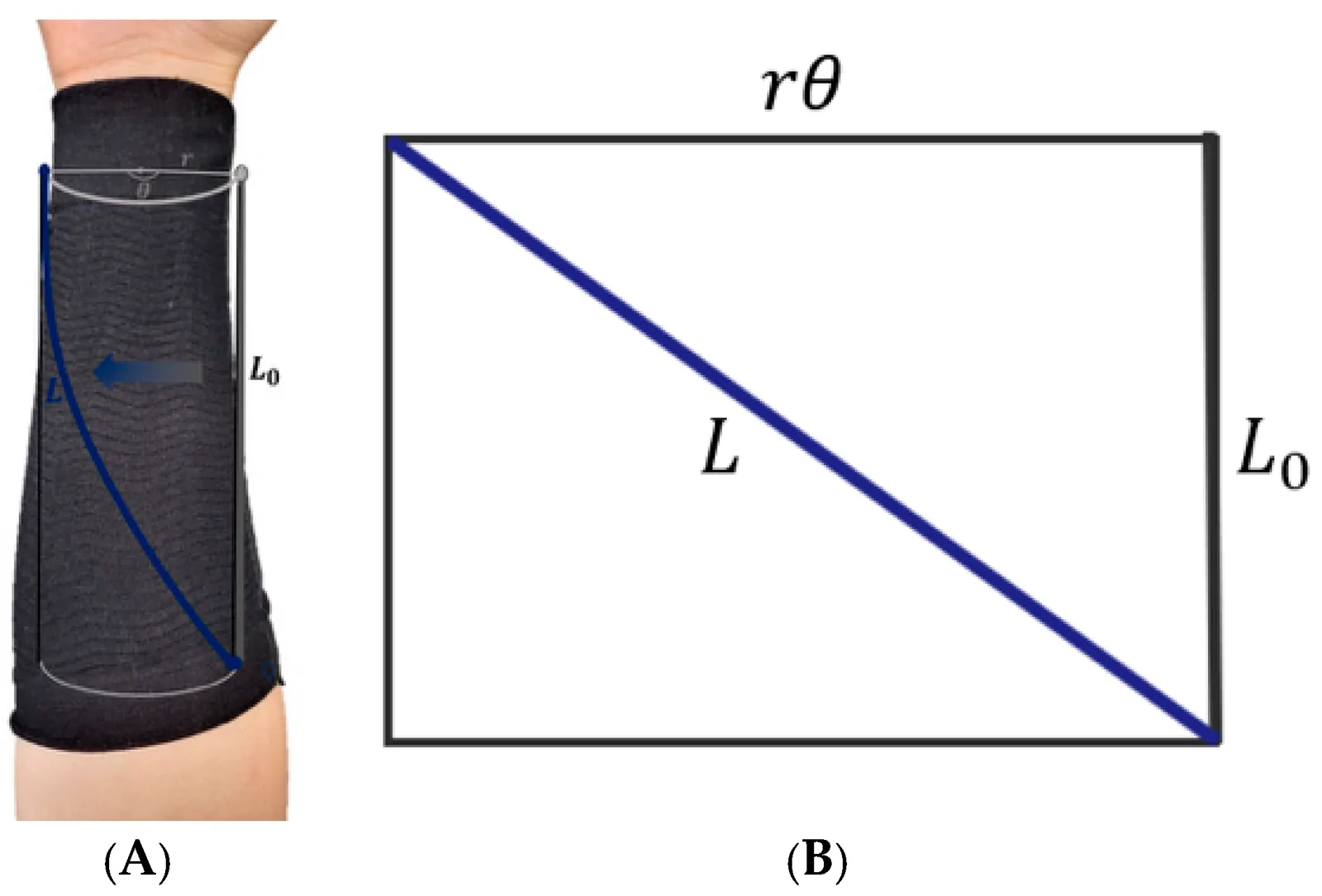
Forearm torsion cylindrical modeling (**A**) and development diagram (**B**).

As illustrated in Figure 5, the Cylinder Model assumes a uniform cylindrical geometry along the forearm link length. Under this assumption, when rotating by θ, the path length of the rotated trajectory is *r*·θ by using the arc length formula. During development, the stretched length can be calculated by the Pythagorean theorem using the path and the initial length. For the value of r, since the forearm is not uniformly cylindrical, an average value for both parts was calculated. The Cylinder Model formulation is given by:

(3) $$L=\sqrt{{\left(\mathrm{r\theta}\right)}^{2}+{L_{0}}^{2}}$$

where L represents the stretched sensor length, *L*_0_ is the initial sensor length, *r* is the average radius of the forearm, and θ represents the forearm rotation angle.

#### 2.3.2. Truncated Cone Model (TCM)

*L*, *L*_0_, and *θ* definitions are the same as the previous model. The development of the truncated cone model is as follows: *R*: Brachioradialis muscle radius, *r*: Wrist radius, *θ_i_*: The central angle, and *L_i_*: The length of the upper arc. 

**Figure 6:**
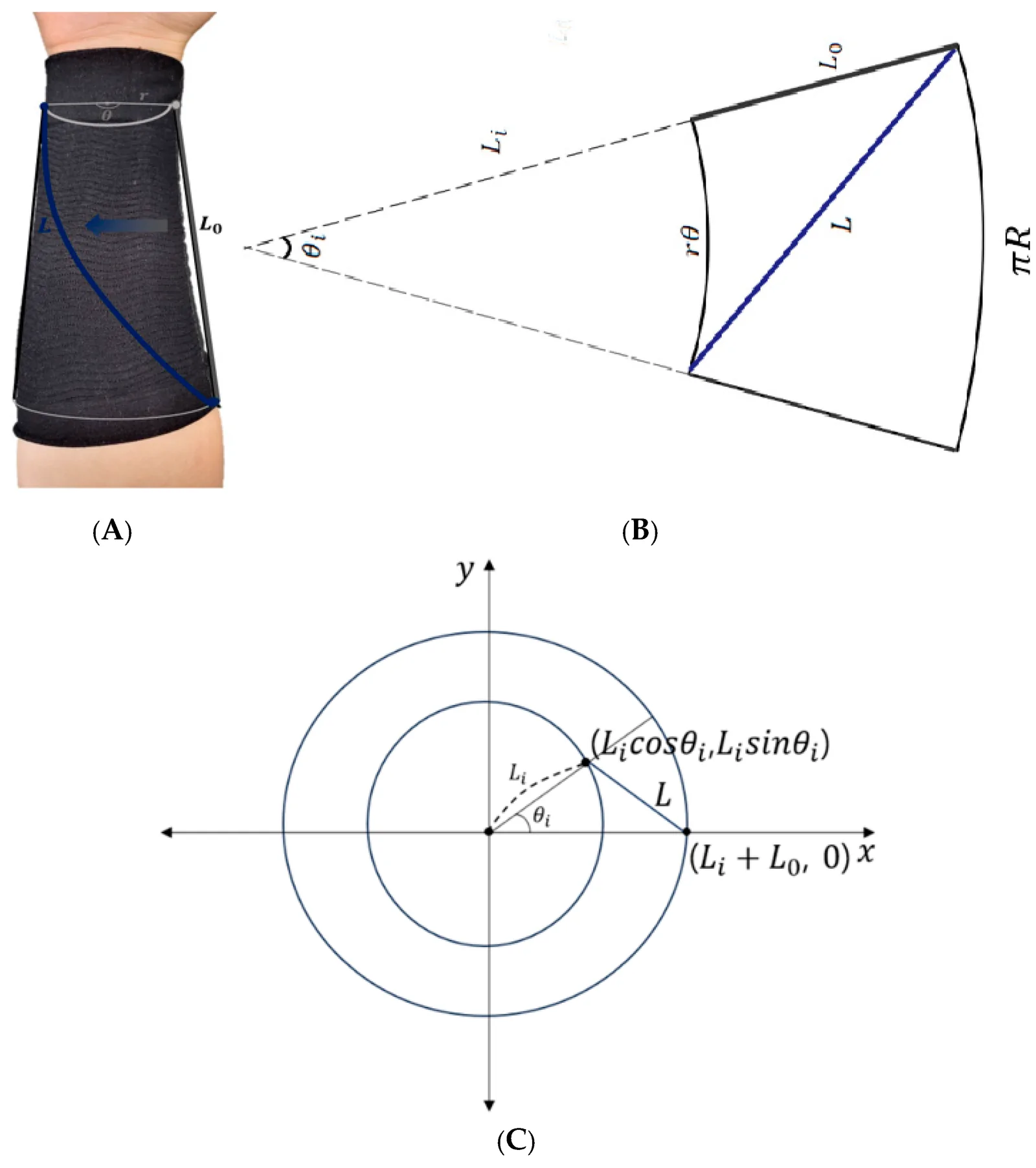
Forearm torsion truncated cone modeling (**A**), development diagram (**B**), and coordinate plot (**C**).

As illustrated in Figure 6, when drawing the trajectory of the truncated cone, *θ_i_* and *L_i_* were defined as the central angle and the radius of the development diagram, respectively. The length of the upper arc is proportional to the radius of the upper arc, as the ratio of the radius of the upper arc to the radius of the lower arc.

*Rθ*:*rθ* = (L*_i_* + L_0_):*L_i_* (4)

By using the arc length formula, the length of the upper arc is determined, thus establishing the relationship between the central angle and the rotation angle.

*L_i_θ_i_* = *rθ* (5)

Using Equations (4) and (5), equations are established for the central angle and the rotation angle. *R* and *L*_0_ represent measured values, whereas *L_i_* is a constant value calculable from Equation (4). Therefore, the central angle is directly proportional to the rotation angle *θ*.

(6) $$\theta_{i}=\frac{R}{\left(L_{i}{+L}_{0}\right)}\theta$$

Subsequently, when calculating the distance between the coordinates at the wrist, which varies with the length, and the fixed coordinates at the brachioradialis muscle, it can be expressed as Equation (7). By inserting the calculated *θ_i_* into the equation, the relationship can be expressed between the rotation angle and the length as an equation.

(7) $$L=\sqrt{{\left(L_{i}+L_{0}-L_{i}\cos\theta_{i}\right)}^{2}+{\left(L_{i}\sin\theta_{i}\right)}^{2}}$$

**Figure 7:**
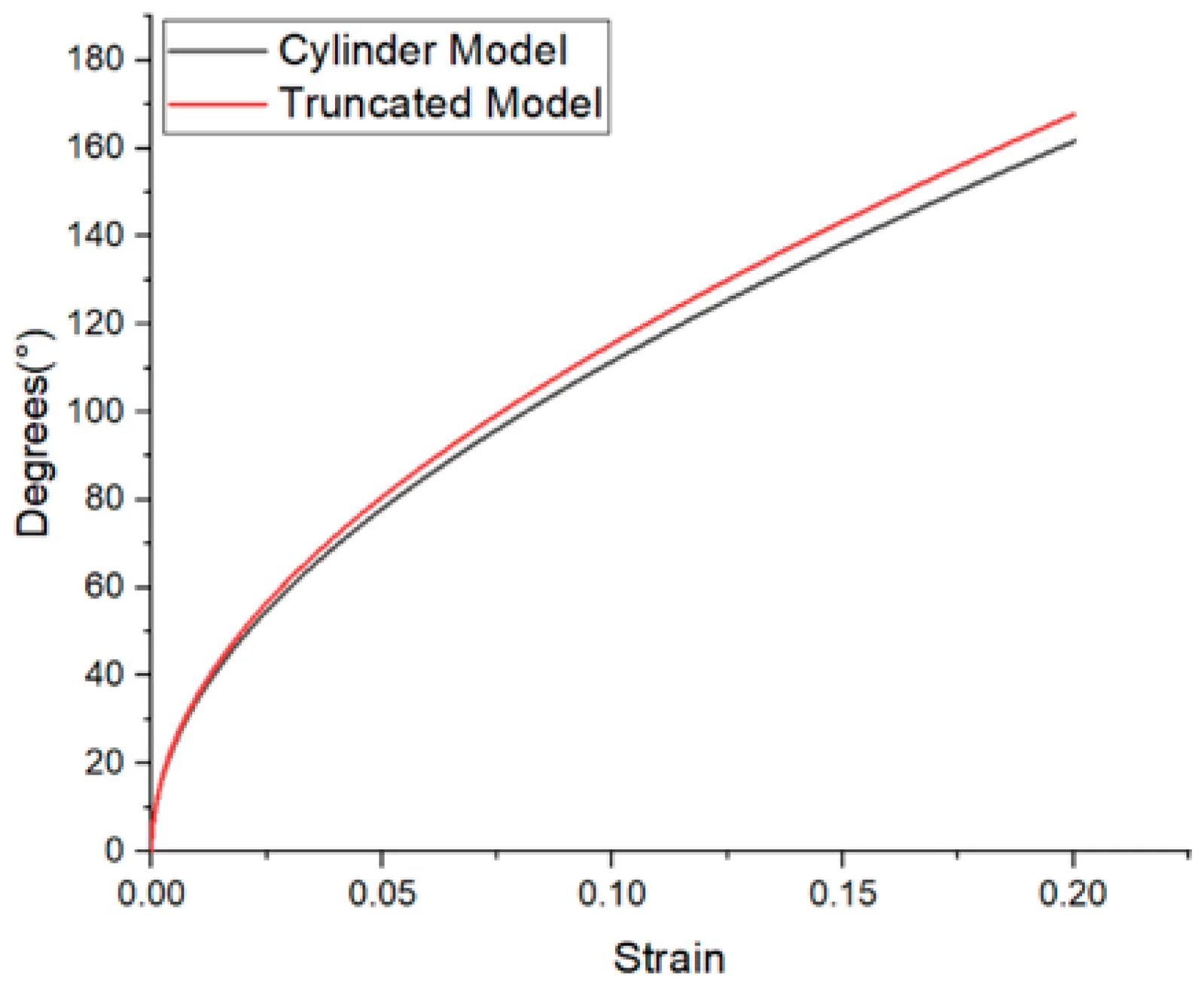
Relationship between rotation angle and estimated strain derived from the geometric models.

The computed *L* through the two models is converted to strain by Equation (2), and the modeling of the relationship between rotation angle and strain is depicted in Figure 7. Using this relationship, the calculated strain values from the relationship between resistance change rate (1) and strain are inserted to calculate the final rotation angle.

### 2.4. Algorithms Architecture and Processing

**Figure 8:**
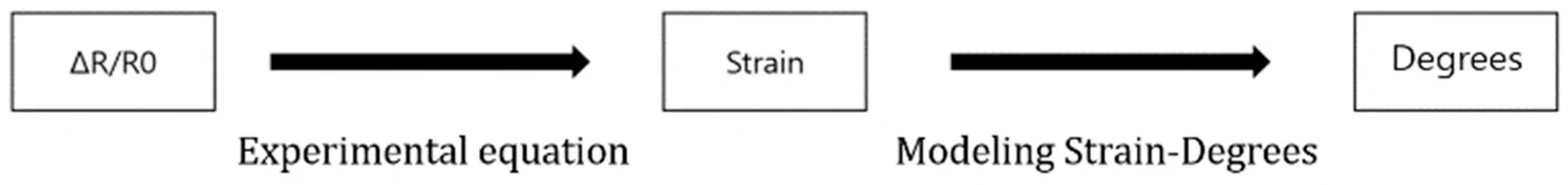
An overall schematic diagram of the torsion sensor algorithm.

The aim of this study was to obtain stretch values through the resistance-strain sensor of a sleeve and convert them into torsion angles using torsion modeling. The processing sequence is summarized in Figure 8. The process is as follows. (1) Raw voltage readings from the strain sensor are acquired via an analog-to-digital converter (ADC) using a voltage divider circuit at a sampling frequency of 10 Hz and converted into instantaneous resistance values. (2) To suppress high-frequency mechanical vibrations, electronic fluctuations, and motion-induced micro-slip artifacts at the skin–sensor interface, raw resistance signals are conditioned using a digital second-order Butterworth low-pass filter with a cutoff frequency of *f_c_* = 1.5 Hz. This cutoff threshold preserves voluntary human forearm rotational dynamics (~0.33 Hz during rapid dynamic trials) and simultaneously suppresses high-frequency contact noise. (3) The relative resistance change rate (*ΔR*/*R*_0_) is converted to tensile strain using the calibrated semi-logarithmic relationship (Equations (1) and (2)). (4) Finally, the strain values are mapped to continuous torsion angles through the analytical kinematic-geometric models (Cylinder or Truncated Cone Model).

Most strain sensors have hysteresis [38,39]. Hysteresis refers to a phenomenon in which the past history of material deformation influences its current state. In simple terms, when it comes to the relationship between strain and resistance change rate, a sensor that has been previously deformed during initial straining should return to its original state with elasticity. However, if sufficient time is not given for it to return to its previous state, it will exhibit a different relationship between strain and resistance change rate during straining compared to the previous state. This hysteresis can lead to inaccurate estimations and reduced estimation accuracy when the sensor is used continuously.

**Figure 9:**
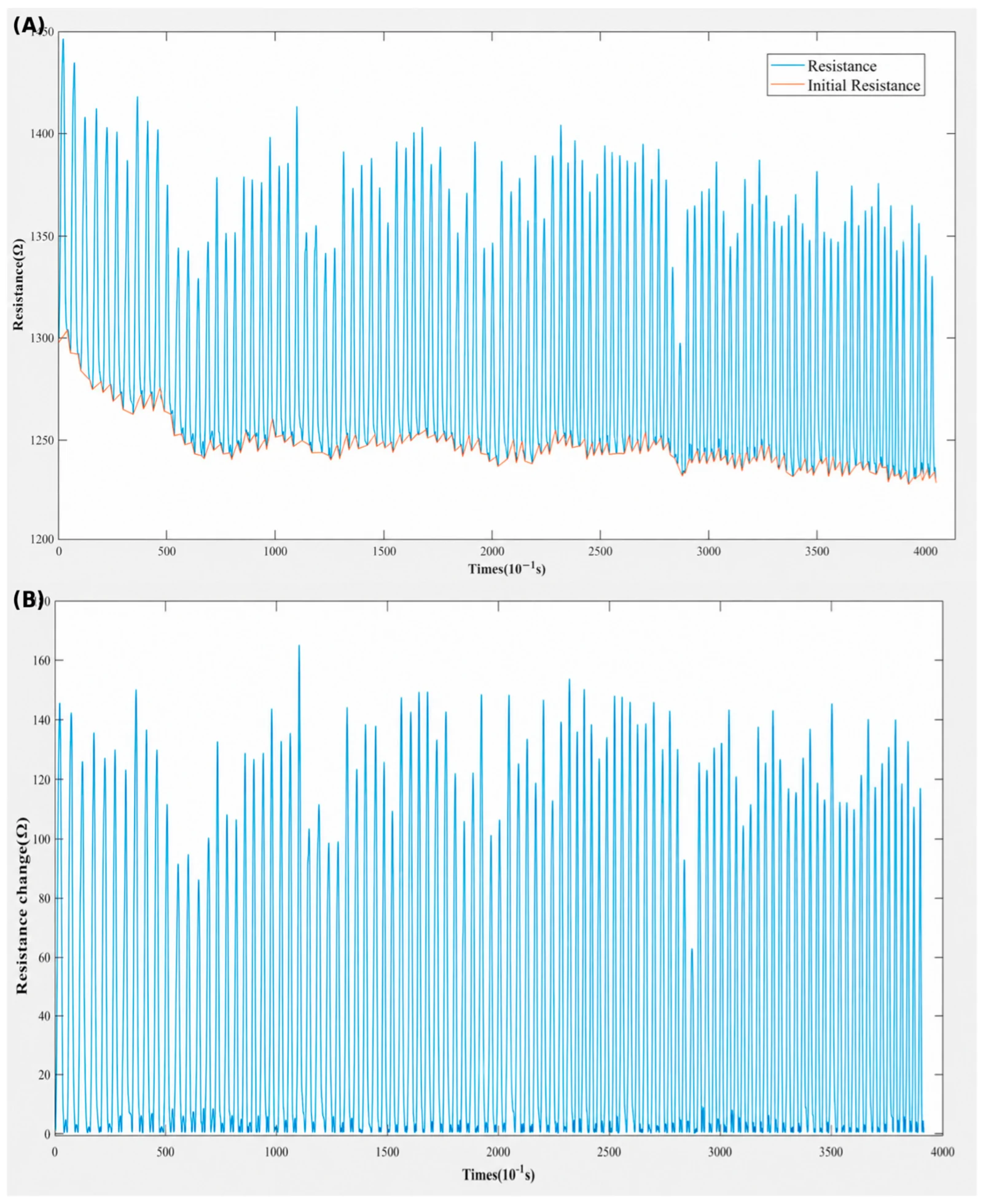
The entire resistance sequence (**A**) and the calculated resistance change values (**B**).

To mitigate baseline drift induced by viscoelastic hysteresis, an online calibration engine (Algorithm 1) was developed to dynamically update the reference baseline for each rotational cycle. As illustrated in Figure 9A, continuous cyclic deformation causes a gradual intra-trial drift in the baseline resistance. To decouple this drift, the filtered resistance sequence was numerically inverted (R_inv = −R_filt) to detect local inflection minima as positive peaks corresponding to cycle boundaries. A piecewise dynamic baseline was then reconstructed across consecutive inflection points via linear interpolation (linspace), which was continuously subtracted from the filtered signal to isolate the true kinematic resistance variation (ΔR_comp). This approach ensures dependable rotation angle estimation across successive cycles irrespective of cumulative material relaxation. Hereafter, the conventional estimation framework without drift compensation is designated as Algorithm A, whereas the dynamic baseline-tracking calibration engine is designated as Algorithm B.

Specifically, the arrival at the initial baseline resistance (*R*_0_) within continuous dynamic cycles is determined by identifying the local minima (troughs) corresponding to the neutral kinematic turning point (0° pronation/supination). In the proposed pipeline, because pure baseline points correspond to inflection minima where the time-derivative of resistance momentarily reaches zero ($\frac{\mathrm{dR}}{\mathrm{dt}}\approx 0$) before subsequent re-stretching, the inverted sequence (R_inv = −R_filt) transforms these troughs into distinct peaks. By enforcing a minimum peak prominence and temporal threshold in FindPeaks, the algorithm robustly confirms the physical neutral starting position of each cycle without confusing transient motion pauses for true mechanical baselines.
**Algorithm 1.** Online Hysteresis-Compensated Calibration Engine (Algorithm B)Input: Filtered resistance sequence R_filt of length N Output: Compensated resistance change ΔR_comp of length N 1: // Step 1: Invert the signal to identify baseline inflection points 2: R_inv ← −R_filt 3: 4: // Step 2: Detect inflection peaks corresponding to cycle boundaries 5: P ← FindPeaks(R_inv)  // P contains indices of the cycle boundaries 6: 7: // Step 3: Piecewise linear interpolation for dynamic baseline tracking 8: Initialize dynamic baseline array B of length N 9: for i = 1 to length(P) − 1 do 10:   idx_start ← P[i] 11:   idx_end  ← P[i + 1] 12:   L_i    ← idx_end − idx_start + 1 13:   B[idx_start : idx_end] ← linspace(R_filt[idx_start], R_filt[idx_end], L_i) 14: end for 15: 16: // Step 4: Calculate the final compensated resistance change 17: ΔR_comp ← R_filt − B 18: 19: RETURN ΔR_comp

### 2.5. Experiment Methods

**Figure 10:**
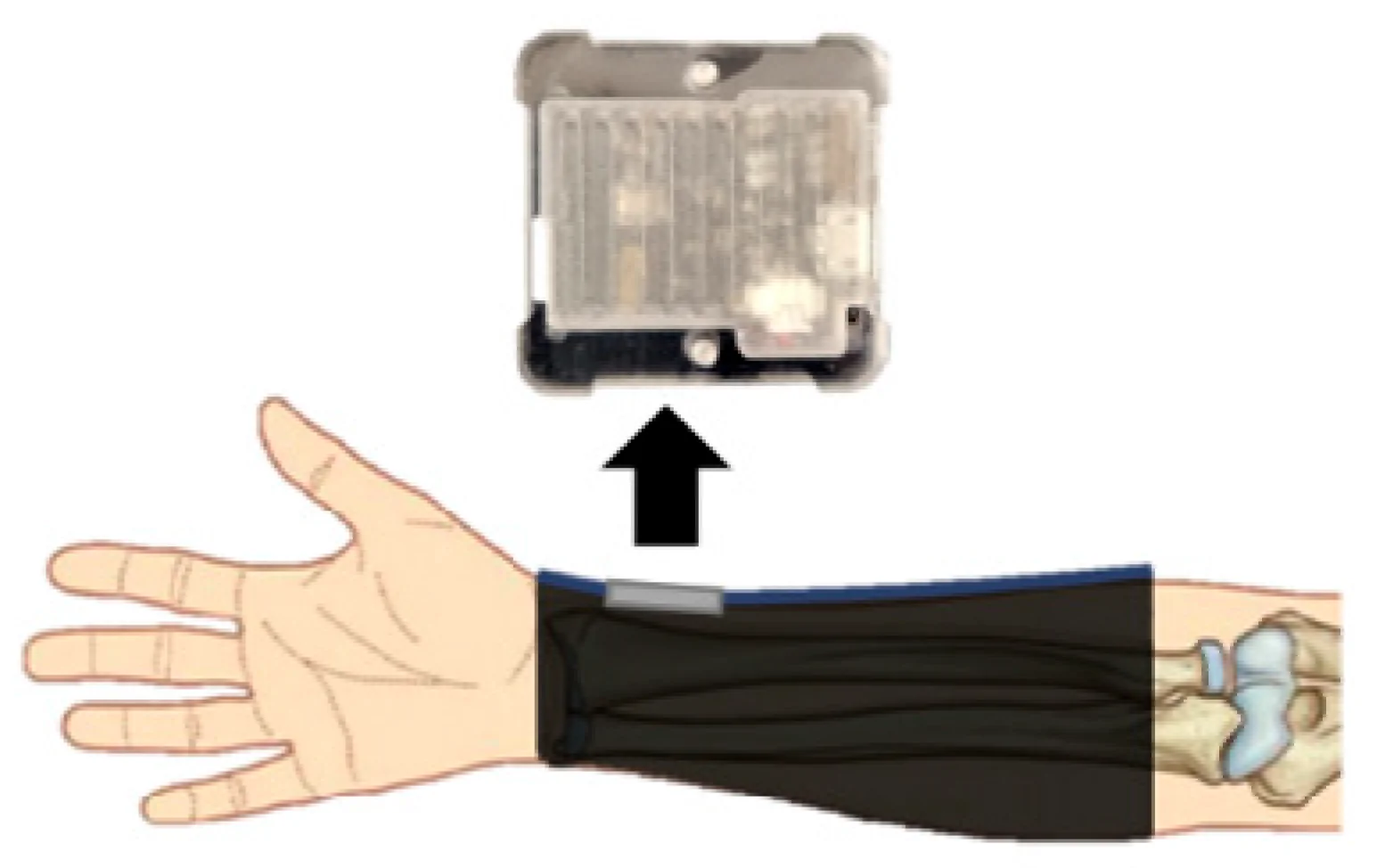
Attachment position of the IMU.

As shown in Figure 10, an IMU (Inertial Measurement Unit) was used as a reference device for measuring the rotation angles [40]. The orientation trajectory was resolved using an onboard Madgwick sensor fusion algorithm, which calculates orientation quaternions by filtering gyroscope angular velocity with accelerometer and magnetometer vectors to suppress orientation drift. The IMU was attached to the external side of the forearm to capture both the sensor torsion and the rotational changes of the IMU simultaneously. Sensor resistance signals were continuously acquired at 10 Hz using the Arduino-based voltage divider circuit configured in Section 2.1. The experiments were conducted under two main conditions: Static and Dynamic situations. In the Static situation, a single torsion movement was performed, taking approximately 14 s to complete the movement slowly. In the Dynamic situation, 20 torsion movements were executed, with each torsion taking about 3 s, performed rapidly. Prior to testing, the conductive rubber cord sensor underwent a mechanical pre-conditioning protocol of 20 continuous loading–unloading cycles up to a strain of ε= 0.25 using the UTM, stabilizing the conductive carbon network and minimizing transient Mullins softening effects. Furthermore, to prevent cumulative viscoelastic creep and thermal stress relaxation across sequential experimental sessions, mandatory rest intervals of at least 5 min were enforced between dynamic trials, allowing the polymer chains to sufficiently recover to their baseline state. Consequently, the intra-trial baseline drift observed during continuous cycling was primarily governed by short-term recovery lags, which Algorithm B effectively decoupled at each inflection point without misinterpreting material hysteresis as true joint motion. In both conditions, the elbow angle was maintained at 0° with the arm fully extended. The anthropometric parameters for the participant (n = 1) were as follows: wrist radius: 3 cm, brachioradialis radius: 5 cm, and the distance between the two joints: 17 cm. These three values were used in the modeling calculations. To ensure millisecond-level time synchronization between the strain sensor and the reference IMU data streams, both devices were sampled through a unified host PC logging architecture utilizing synchronized hardware timestamps, and the signals were temporally aligned by identifying the onset inflection marker of the initial rotational cycle.

### 2.6. Statistical Analysis

To rigorously assess the performance improvements yielded by the geometric modeling and online drift calibration engine, non-parametric statistical analyses were conducted on the cycle-by-cycle error distributions across the 20 dynamic rotation cycles. Non-parametric approaches were selected because kinematic peak error distributions often violate the normality assumption in wearable movement tracking. For multiple condition comparisons (evaluating downstream performance across Algorithm A vs. Algorithm B and Cylinder vs. Truncated Cone Model), a Friedman test was conducted [41]. For pairwise evaluations between paired cycle distributions—specifically comparing uncompensated (Algorithm A) and hysteresis-compensated (Algorithm B) estimation within each model, as well as comparing the Cylinder Model to the Truncated Cone Model under Algorithm B—the Wilcoxon signed-rank test was utilized [42]. For all statistical evaluations, the significance threshold was set at *p* < 0.05.

## 3. Results

### 3.1. Estimation of Torsion Angle in Static Motion

#### 3.1.1. Analysis of Modeling Accuracy

**Figure 11:**
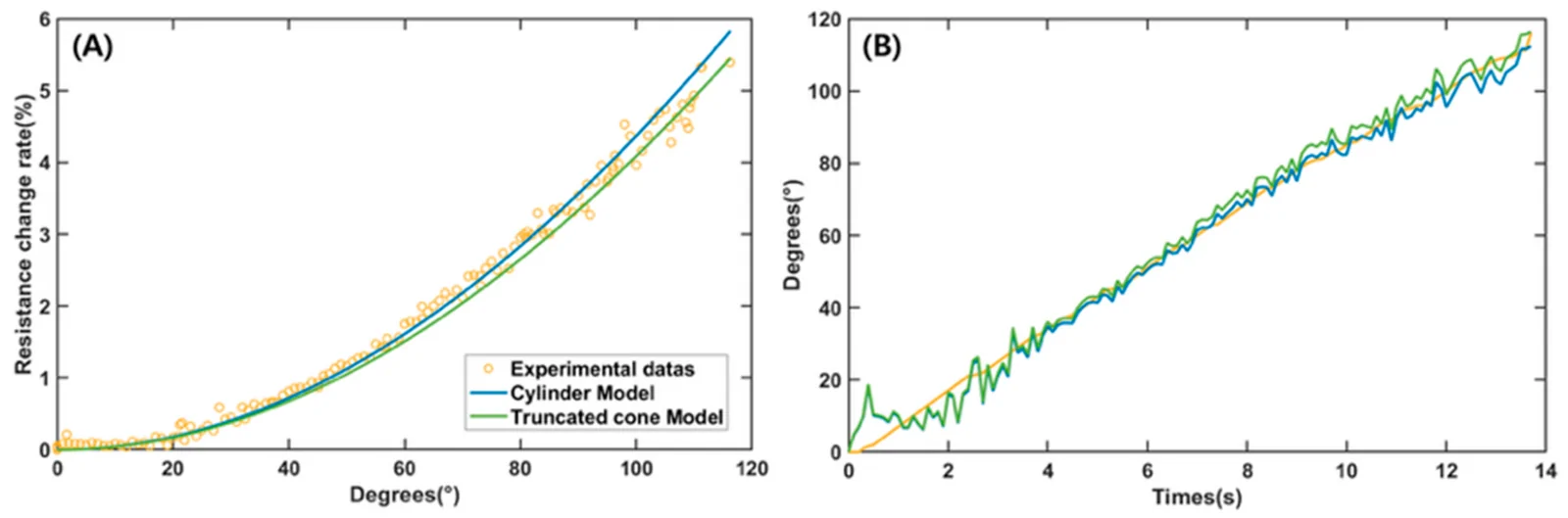
Torsion angle estimation analysis in static motion. (**A**) Degree-resistance rate graph for assessing model accuracy. (**B**) Time-angle graph comparing estimated and experimental values.

Before comparing estimated angles, an accuracy assessment was conducted between the modeling and experimental values using the reference equipment. As depicted in Figure 11A, resistance rate changes with respect to the rotation angle for both modeling approaches were plotted and compared with experimental values. To gather these specific data, the static experimental procedure described in Section 2.5 was executed: a single forearm torsion movement was performed slowly over approximately 14 s while maintaining the elbow extended at 0°. The ground truth rotation angles were continuously captured by the IMU, strictly time-synchronized with the resistance changes sampled at 10 Hz via the voltage divider circuit.

As summarized in Table 1, the comparison results showed a high level of similarity, approximately 0.9925, for both the Cylinder and Truncated Cone Models. In previous research, a regression analysis of angle-resistance-tension sensor voltage obtained through six experiments was compared with the actual measured values, resulting in a determination coefficient of 0.9802 in pronation motion and 0.9985 in supination motion [33]. These results indicate that the present proof-of-concept measurements achieved a level of agreement comparable to that reported in previous studies, although direct comparison should be interpreted cautiously because of differences in experimental protocols and study scope.

#### 3.1.2. Analysis of the Accuracy of the Time-Angle Sequence in Static Motion

The relationship was examined between the estimated angle and the IMU rotation angle in the Static condition. In contrast to previous research, instead of confirming accuracy with only a few experimental values, the estimation accuracy was verified by comparing the continuously changing angle values over time, as illustrated in Figure 11B. Both estimated values exhibited equally high accuracy, with a determination coefficient of 0.9888. In a Static condition, the impact of hysteresis is minimal, so the results were successfully derived by simply setting the initial resistance value without the need for algorithmic correction processes.

### 3.2. Estimation of Torsion Angle in Dynamic Motion

#### 3.2.1. Analysis of the Accuracy of the Time-Angle Sequence in Dynamic Motion

**Figure 12:**
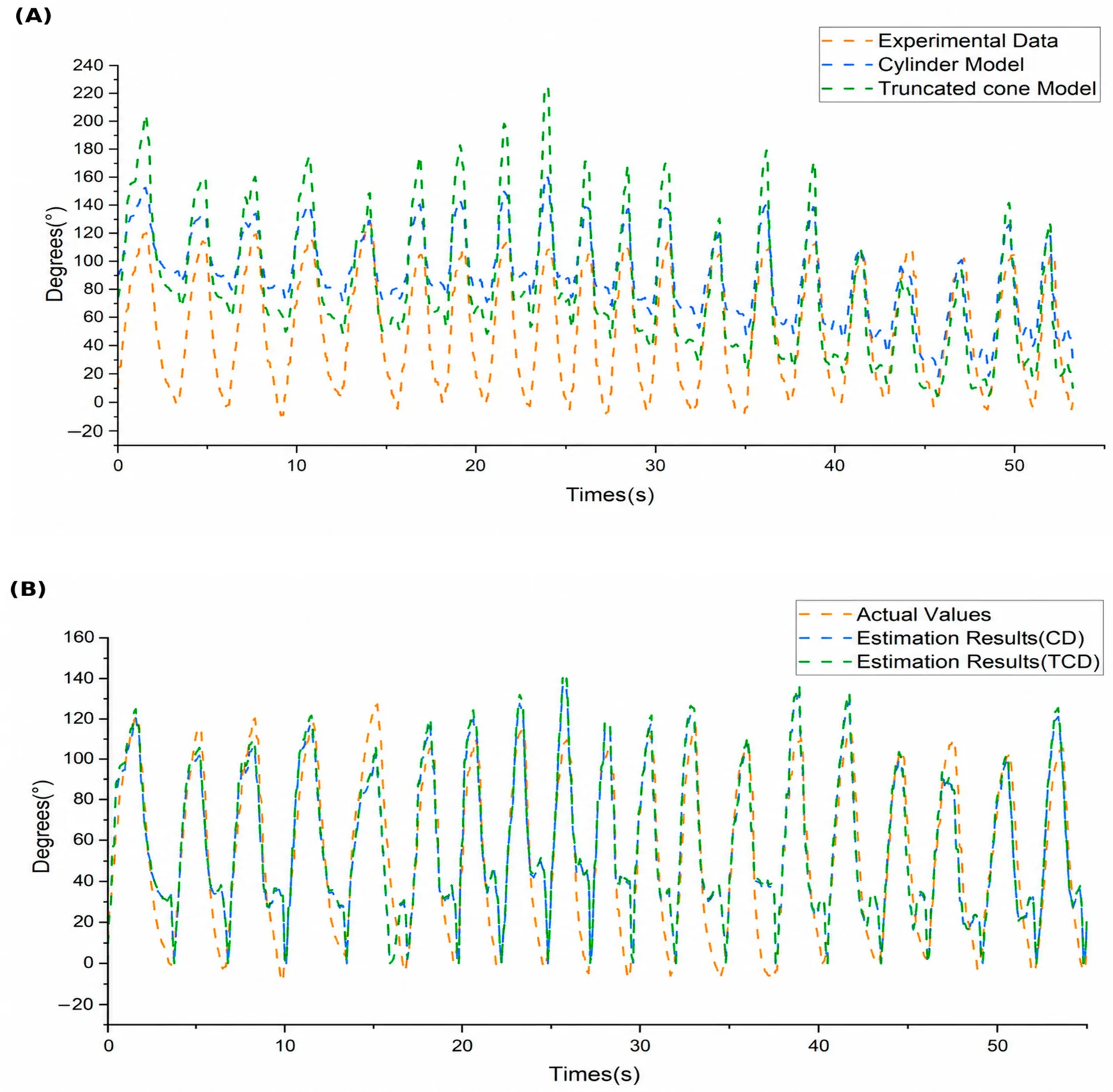
Time-angle graphs in Dynamic motion. (**A**) Algorithm without hysteresis correction. (**B**) Algorithm with hysteresis correction. Note: The legend provided in the first subplot applies uniformly across all subplots to optimize space.

Figure 12A illustrates the continuous rotation angles estimated via Algorithm A (without hysteresis compensation), whereas Figure 12B presents the dynamic trajectory estimated via Algorithm B alongside the reference IMU data. As shown in the time-series profiles, uncompensated tracking (Figure 12A) exhibits a pronounced downward baseline drift and trajectory degradation over time, diverging substantially from the actual experimental values. In contrast, the hysteresis-compensated framework (Figure 12B) demonstrates that the estimated angles closely and stably follow the reference motion profile across dynamic cycles. Under the uncompensated condition (Algorithm A), the Truncated Cone Model achieved a substantially higher coefficient of determination (*R*^2^ = 0.7811) compared to the Cylinder Model (*R*^2^ = 0.5760), demonstrating that accounting for anatomical tapering provides superior geometric fidelity even under severe baseline drift. When hysteresis compensation was applied (Algorithm B), both geometric representations maintained robust and comparable tracking performance (*R*^2^ ≈ 0.8033, Table 2). Overall, the visual trajectory and correlation analyses indicate that Algorithm B with online hysteresis correction substantially improved dynamic torsion estimation under the present experimental conditions.

#### 3.2.2. Evaluation of Algorithms Through Estimated Value Analysis

**Figure 13:**
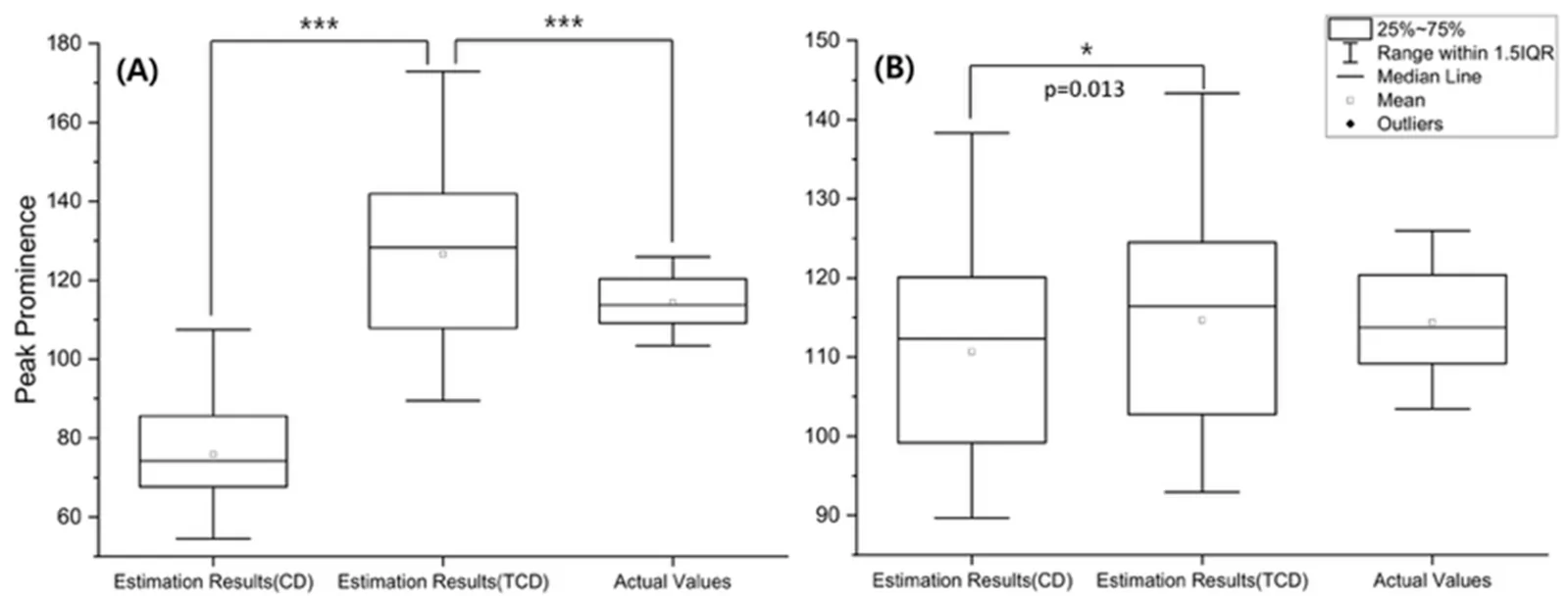
Torsion quantity box charts for Algorithm A (**A**) and Algorithm B (**B**). Asterisks indicate statistically significant differences based on Wilcoxon signed-rank tests (* *p* < 0.05, *** *p* < 0.001).

Peak prominence was evaluated to quantify the dynamic rotational amplitude, defined as the angular difference between the baseline trough and the maximum positive peak within each cycle. For Algorithm A, the average peak prominence for the Cylinder Model (CM, designated as CD in Table 3 and Figure 12 and Figure 13) was 75.87°, the Truncated Cone Model (TCM, designated as TCD) was 126.70°, and the experimental value was 114.38°, indicating that Algorithm A underestimated the rotational extent for the Cylinder Model but overestimated it for the Truncated Cone Model, with the Truncated Cone Model exhibiting wider cycle fluctuations. In contrast, under Algorithm B, the average peak prominence was 110.66° for the Cylinder Model and 114.67° for the Truncated Cone Model, closely aligning with the experimental reference value of 114.39°. Overall, these results indicate that Algorithm B provides improved fidelity in tracking torsional amplitude under the present experimental conditions. In terms of cycle-by-cycle relative error (RE), Algorithm A exhibited a large prominence error of 48.33% for the Truncated Cone Model due to uncompensated baseline drift, whereas Algorithm B dramatically reduced this error to just 0.42%. 

The positive peak represents the absolute value reached during the maximum rotation. In terms of the final rotation angle (positive peaks), the Truncated Cone Model exhibited a markedly lower relative error (18.80%) than the Cylinder Model (33.46%) under Algorithm A. Under Algorithm B, both geometric models yielded comparable high accuracy, with the Cylinder Model achieving 10.21% and the Truncated Cone Model achieving 11.58% (differing by only 1.37 percentage points). Without hysteresis correction, the Truncated Cone Model appears to be more suitable for estimating the final angle, whereas the Cylinder Model is more suitable for estimating the extent of torsion. With hysteresis correction, both models demonstrated comparable absolute peak tracking, but the Truncated Cone Model significantly reduced cycle-to-cycle error dispersion (interquartile range in Figure 13B). In terms of absolute angular tracking precision, Algorithm B substantially reduced the root-mean-square error (RMSE) from 19.42° down to 6.84° for the Cylinder Model, and from 15.68° down to 4.12° for the Truncated Cone Model, demonstrating superior trajectory tracking fidelity. The multi-condition Friedman test confirmed a statistically significant difference across the four algorithm-model configurations (*χ*^2^(3) = 48.62, *p* < 0.001). Subsequent pairwise Wilcoxon signed-rank tests confirmed that Algorithm B achieved a statistically significant error reduction over Algorithm A for both the Cylinder Model (Z = −3.92, *p* < 0.001) and the Truncated Cone Model (Z = −3.92, *p* < 0.001). Furthermore, within the hysteresis-compensated condition (Algorithm B), the Truncated Cone Model yielded significantly lower cycle-to-cycle dispersion and peak prominence error compared to the Cylinder Model (Z = −2.48, *p* = 0.013). These results suggest that among the evaluated configurations, the hysteresis-corrected Truncated Cone Model provided the most favorable torsion-angle estimation performance under the present experimental conditions.

## 4. Discussion

The proposed kinematic-geometric framework combined with an online calibration engine demonstrates a robust, training-free method for measuring multi-axial torsional movements using soft resistive strain sensors. Unlike prior approaches that heavily depend on empirical polynomial fitting or data-intensive deep learning models, the proposed approach establishes direct structural causality between non-linear sensor deformation and rotation angles.

### 4.1. Comparison of Geometric Models: Cylinder vs. Truncated Cone Model

Under static conditions, both the Cylinder Model (CM) and Truncated Cone Model (TCM) achieved an exceptionally high coefficient of determination (R^2^ = 0.9888) against the reference IMU data. This indicates that when structural deformation occurs slowly and viscoelastic recovery delay is negligible, basic geometric representations accurately map tensile strain to torsional rotation.

However, under dynamic conditions, clear performance discrepancies emerge between the two models depending on whether hysteresis compensation is applied. In Algorithm A (uncompensated), the Truncated Cone Model exhibited a significantly lower relative error in predicting positive peaks (18.80%) compared to the Cylinder Model (33.46%). This improvement stems from the ability of the TCM to account for the spatial gradient in forearm radius (tapering from the brachioradialis muscle to the wrist).

When combined with hysteresis correction (Algorithm B), both models demonstrated a comparable overall correlation (R^2^ ≈ 0.8033). Crucially, however, the TCM achieved an angular RMSE of 4.12° (reduced from 15.68°) alongside a cycle peak prominence relative error of 0.42% and significantly reduced cycle-to-cycle error dispersion (interquartile range in Figure 13B). These results suggest that accounting for the tapered conical structure of the rotating body improves the representation of cycle-to-cycle amplitude variations and geometric changes during rapid motion under the present experimental conditions.

### 4.2. Efficacy of the Hysteresis-Compensated Calibration Engine

Viscoelastic hysteresis and baseline drift are well-known limitations of elastomeric soft sensors during cyclic dynamic deformation. As demonstrated in Figure 9A and Figure 12A, uncompensated sensor data led to cumulative trajectory drift, causing Algorithm A to continuously underestimate or misalign torsional peak positions (R^2^ = 0.5760 for CM, 0.7811 for TCM).

Algorithm B effectively mitigates this issue by dynamically tracking initial resistance coordinates at each inflection point and establishing an online baseline reference via piecewise linear interpolation. This real-time drift cancellation dramatically improved tracking performance (R^2^ = 0.8033) and reduced peak prominence error from 48.33% down to 0.42% for the TCM. Because this calibration engine relies on lightweight analytical functions (linspace interpolation and peak identification) rather than heavy computational optimization, it is computationally lightweight and may be suitable for real-time embedded implementation.

### 4.3. Limitations and Environmental Vulnerabilities

Despite its high accuracy and physical interpretability, several limitations must be noted:

1. Motion Artifacts and Cross-Axis Parasitic Coupling: While attaching the sensor along the ulnar side minimizes direct muscle belly bulge interference, compound forearm movements involving active isometric gripping or simultaneous elbow flexion can still induce cross-axis radial strain. Such volumetric muscle expansion acts as parasitic cross-talk that superimposes onto the kinematic torsional strain. In the current study, this interference was minimized by maintaining full elbow extension (0^°^) and a relaxed hand posture during the experimental trials. However, in free-living activities or loaded ergonomic tasks, integrating an orthogonal strain channel or an auxiliary circumferential band to directly quantify radial volumetric expansion will be necessary to analytically subtract muscle bulge artifacts from pure torsional shear deformation.
2. Single-Subject Feasibility Evaluation and Morphological Constraints: The proposed geometric models rely on three static anthropometric inputs (*r*, *R*, and *L*_0_). However, this study was conducted as a single-subject proof-of-concept feasibility study (n = 1) under controlled laboratory conditions and therefore does not constitute population-level or clinical validation. Because soft tissue compliance, subcutaneous fat thickness, and sleeve-skin shear interactions vary across individuals, the universal transferability of these closed-form formulations remains to be fully established. Future investigations involving multiple participants with diverse anthropometric and anatomical characteristics are required to establish the generalizability of the framework across broader populations.
3. Sampling Frequency Constraints: The experimental setup utilized a 10 Hz sampling rate via Arduino Uno. Although sufficient for routine human movements (~3 s per turn), ultra-fast dynamic movements or high-frequency robotic oscillations will require higher acquisition rates and adaptive peak-detection thresholding.
4. Baseline Drift and Limitations of Resistive Sensing: As illustrated in Figure 9A, the conductive rubber cord sensor exhibits cumulative baseline drift and viscoelastic recovery lags across dynamic cycles, which represents an intrinsic limitation of elastomeric resistive strain sensors. Although the proposed Algorithm B executes dynamic inflection-point tracking to cancel drift in a computationally efficient, online cycle-to-cycle framework, the sensor signal still requires algorithmic offset re-anchoring at rotational turning points rather than yielding an intrinsically stable absolute resistance. This operating characteristic can introduce minor latency in continuous real-time closed-loop control during aperiodic or non-cyclic movements. To fundamentally overcome the unstable baseline and hysteretic lag of resistive elastomeric composites, advanced capacitive flexible strain sensing architectures present a promising alternative. In particular, planar capacitive interdigital electrodes (IDEs) with structural micro-engineering have demonstrated outstanding baseline stability, negligible hysteresis, and direct strain reversibility without necessitating computational baseline correction or offline post-processing [43]. Incorporating such drift-free capacitive IDE architectures into wearable sleeves constitutes a key direction for future work to achieve direct, single-stage kinematic tracking.

## 5. Conclusions

This paper presented a training-free torsional motion tracking framework by integrating analytical kinematic-geometric modeling with a hysteresis-compensated calibration engine.

- Static Accuracy: The geometric mapping models (CM and TCM) achieved R^2^ = 0.9888 correlation with multi-axis IMU ground truth during static rotation.
- Dynamic Robustness: The online hysteresis correction algorithm (Algorithm B) substantially reduced the effects of baseline drift inherent to elastomeric strain sensors, improving dynamic tracking fidelity and reducing the cycle peak prominence error to 0.42%.
- Model Suitability: Under Algorithm B, the Truncated Cone Model achieved an angular RMSE of 4.12° (with a cycle peak prominence relative error of 0.42%), demonstrating superior structural fidelity compared to the cylindrical approximation. Based on evaluation with a single participant (n = 1), this proof-of-concept study demonstrates the technical feasibility of training-free torsional tracking while emphasizing the need for future multi-participant evaluations before broader human application.

Future Outlook: While the hysteresis-compensated Truncated Cone Model achieved an angular RMSE of 4.12° (and a peak prominence error of 0.42%), it is crucial to note appropriate caveats regarding these laboratory-constrained experimental conditions. To prevent over-claiming performance for unconstrained real-world scenarios, future studies must evaluate the robustness of the framework against complex, free-living motion artifacts. Because the proposed framework is mathematically explicit and training-free, it can in principle be adapted to different limb sizes or soft robotic joints by updating the basic physical boundary dimensions (*r*, *R*, *L*_0_). However, its performance across different human anatomical profiles remains to be experimentally established through multi-participant studies. To broaden the application scope, preliminary feasibility testing on soft-robot torsion joints—specifically embedding this sensing strategy into the outer skin of twisted-and-coiled polymer actuators (TCPAs) and pneumatic artificial muscles (PAMs)—will be a primary focus in future work. Furthermore, to eliminate the reliance on algorithmic baseline re-anchoring necessitated by resistive hysteresis, future iterations will explore the integration of planar capacitive interdigital electrodes (IDEs), enabling drift-free, direct capacitive strain acquisition for high-speed, uncalibrated soft robotic closed-loop control.

## Figures and Tables

**Table 1:** Coefficient of determination for accuracy analysis by model.

Modeling	Cylinder	Truncated Cone
R-squared	0.99251	0.99253

**Table 2:** Coefficient of determination for algorithms and models in Dynamic motion.

Modeling	Cylinder	Truncated Cone
R-squared of algorithm A	0.57603	0.78114
R-squared of algorithm B	0.80325	0.80328

**Table 3:** Relative error (RE, %) and angular root-mean-square error (RMSE, °) between the estimated and actual rotation angles per cycle for Algorithm A and Algorithm B.

**Algorithm A**	**CD**	**TCD**
RE of peak prominences	22.08	48.33
RE of positive peaks	33.46	18.80
Angular RMSE (°)	19.42	15.68
**Algorithm B**	**CD**	**TCD**
RE of peak prominences	3.09	0.42
RE of positive peaks	10.21	11.58
Angular RMSE (°)	6.84	4.12

## Data Availability

The data presented in this study are available on reasonable request from the corresponding author.

## References

[1] Liu W., Long Z., Yang G., Xing L. (2022). A self-powered wearable motion sensor for monitoring volleyball skill and building big sports data. Biosensors.

[2] Kos M., Kramberger I. (2017). A wearable device and system for movement and biometric data acquisition for sports applications. IEEE Access.

[3] Park S.-H., Yi J., Kim D., Lee Y., Koo H.S., Park Y.-L. (2019). A lightweight, soft wearable sleeve for rehabilitation of forearm pronation and supination. Proceedings of the 2019 2nd IEEE International Conference on Soft Robotics (RoboSoft), Seoul, Republic of Korea, 14–18 April 2019.

[4] Suglia V., Camardella C., Rinaldi G., Chiaradia D., Buongiorno D., Leonardis D., Zhou H., Frisoli A., Bevilacqua V. (2026). Muscle networks analysis on an active occupational shoulder exoskeleton. Biomed. Signal Process. Control.

[5] Ghislieri M., Locoratolo L., Sciscenti F., Lanotte M., Rizzi L., Agostini V. (2026). Muscle Network of Parkinson’s Gait: A 12-Month Longitudinal Analysis Before and After Deep Brain Stimulation Surgery. IEEE Trans. Neural Syst. Rehabil. Eng..

[6] Scano A., Chiavenna A., Caimmi M., Malosio M., Tosatti L.M., Molteni F. (2017). Effect of human-robot interaction on muscular synergies on healthy people and post-stroke chronic patients. Proceedings of the 2017 International Conference on Rehabilitation Robotics (ICORR), London, UK, 17–20 July 2017.

[7] Pan B., Liu T. (2023). Coherence networks of bilateral upper limb motions in chronic stroke patients. J. Mech. Med. Biol..

[8] Zhao K., Feng Y., Li L., Zhou Y., Zhang Z., Li J. (2024). Muscle synergies and muscle networks in multiple frequency components in post-stroke patients. Biomed. Signal Process. Control.

[9] Suglia V., Bonab A.K., Leonardis D., Bevilacqua V., Frisoli A., Chiaradia D., Camardella C. (2026). Muscle Coordination during an Endurance Walking Task With Elbow Exosuit Assistance. IEEE Trans. Neural Syst. Rehabil. Eng..

[10] Bonab A.K., Camardella C., Frisoli A., Chiaradia D. (2025). Muscle Synergy Analysis of Healthy Subjects Using a Soft Elbow Exosuit During Load-Carrying Tasks. Proceedings of the 2025 International Conference On Rehabilitation Robotics (ICORR), Chicago, IL, USA, 12–16 May 2025.

[11] Bangaru S.S., Wang C., Aghazadeh F. (2022). Automated and continuous fatigue monitoring in construction workers using forearm EMG and IMU wearable sensors and recurrent neural network. Sensors.

[12] Chun S., Kim S., Kim J. (2023). Human arm workout classification by arm sleeve device based on machine learning algorithms. Sensors.

[13] Koo B., Nguyen N.T., Kim J. (2023). Identification and classification of human body exercises on smart textile bands by combining decision tree and convolutional neural networks. Sensors.

[14] Favata A., Gallart-Agut R., Pamies-Vila R., Torras C., Font-Llagunes J.M. (2024). IMU-based systems for upper-limb kinematic analysis in clinical applications: A systematic review. IEEE Sens. J..

[15] Amjadi M., Kyung K.U., Park I., Sitti M. (2016). Stretchable, skin-mountable, and wearable strain sensors and their potential applications: A review. Adv. Funct. Mater..

[16] Vu C.C., Nguyen T.T., Kim S., Kim J. (2021). Effects of 3D printing-line directions for stretchable sensor performances. Materials.

[17] Ryu S., Lee P., Chou J.B., Xu R., Zhao R., Hart A.J., Kim S.-G. (2015). Extremely elastic wearable carbon nanotube fiber strain sensor for monitoring of human motion. ACS Nano.

[18] Amjadi M., Turan M., Clementson C.P., Sitti M. (2016). Parallel microcracks-based ultrasensitive and highly stretchable strain sensors. ACS Appl. Mater. Interfaces.

[19] Vu C.C., Kim J. (2018). Human motion recognition by textile sensors based on machine learning algorithms. Sensors.

[20] Vu C.C., Kim J. (2020). Highly sensitive e-textile strain sensors enhanced by geometrical treatment for human monitoring. Sensors.

[21] Souri H., Banerjee H., Jusufi A., Radacsi N., Stokes A.A., Park I., Sitti M., Amjadi M. (2020). Wearable and stretchable strain sensors: Materials, sensing mechanisms, and applications. Adv. Intell. Syst..

[22] Kim J., Kim J. (2023). Classification of breathing signals according to human motions by combining 1D convolutional neural network and embroidered textile sensor. Sensors.

[23] Biswas D., Cranny A., Gupta N., Maharatna K., Achner J., Klemke J., Jöbges M., Ortmann S. (2015). Recognizing upper limb movements with wrist worn inertial sensors using k-means clustering classification. Hum. Mov. Sci..

[24] Li C., Liu D., Xu C., Wang Z., Shu S., Sun Z., Tang W., Wang Z.L. (2021). Sensing of joint and spinal bending or stretching via a retractable and wearable badge reel. Nat. Commun..

[25] Saucier D.N., Davarzani S., Burch V R.F., Chander H., Strawderman L., Freeman C., Ogden L., Petway A., Duvall A., Crane C. (2021). External load and muscle activation monitoring of NCAA Division I basketball team using smart compression shorts. Sensors.

[26] Lai J., Zhou H., Jin Z., Li S., Liu H., Jin X., Luo C., Ma A., Chen W. (2019). Highly stretchable, fatigue-resistant, electrically conductive, and temperature-tolerant ionogels for high-performance flexible sensors. ACS Appl. Mater. Interfaces.

[27] Wang P., Li G., Yu W., Meng C., Guo S. (2022). Color-customizable, stretchable, self-healable and degradable ionic gel for variable human-motion detection via strain, pressure, and torsion. Adv. Mater. Interfaces.

[28] Nakamoto H., Ootaka H., Tada M., Hirata I., Kobayashi F., Kojima F. (2016). Stretchable strain sensor with anisotropy and application for joint angle measurement. IEEE Sens. J..

[29] Park J.W., Kim T., Kim D., Hong Y., Gong H.S. (2019). Measurement of finger joint angle using stretchable carbon nanotube strain sensor. PLoS ONE.

[30] Gholami M., Ejupi A., Rezaei A., Ferrone A., Menon C. (2018). Estimation of knee joint angle using a fabric-based strain sensor and machine learning: A preliminary investigation. Proceedings of the 2018 7th IEEE International Conference on Biomedical Robotics and Biomechatronics (Biorob), Enschede, The Netherlands, 26–29 August 2018.

[31] Wang J., Lu C., Zhang K. (2020). Textile-based strain sensor for human motion detection. Energy Environ. Mater..

[32] Zhang M., Wang M., Zhang M., Gao Q., Feng X., Zhang Y., Hu J., Wu G. (2020). Stretchable conductive Ni@ Fe_3_O_4_@ Polyester fabric strain sensor with negative resistance variation and electromagnetic interference shielding. Org. Electron..

[33] Huang B., Li M., Mei T., McCoul D., Qin S., Zhao Z., Zhao J. (2017). Wearable stretch sensors for motion measurement of the wrist joint based on dielectric elastomers. Sensors.

[34] Cha Y., Chung J., Hur S.-M. (2019). Torsion sensing on a cylinder using a flexible piezoelectric wrist band. IEEE/ASME Trans. Mechatron..

[35] Matsuoka J., Berger R.A., Berglund L.J., An K.-N. (2006). An analysis of symmetry of torque strength of the forearm under resisted forearm rotation in normal subjects. J. Hand Surg..

[36] Zarzycka N., Załuska S. (1989). Measurements of the forearm i inhabitants of the Lublin region. Annales Universitatis Mariae Curie-Sklodowska.

[37] Yang S., Lu N. (2013). Gauge factor and stretchability of silicon-on-polymer strain gauges. Sensors.

[38] Guo L., Berglin L., Mattila H. (2010). Textile strain sensors characterization-sensitivity, linearity, stability and hysteresis. Nord. Text. J..

[39] Mandolino M.A., Scholtes D., Ferrante F., Rizzello G. (2023). A physics-based hybrid dynamical model of hysteresis in polycrystalline shape memory alloy wire transducers. IEEE/Asme Trans. Mechatron..

[40] Meng Y., Fang G., Yang J., Guo Y., Wang C.C. (2023). Spring-IMU fusion-based proprioception for feedback control of soft manipulators. IEEE/ASME Trans. Mechatron..

[41] Friedman M. (1937). The use of ranks to avoid the assumption of normality implicit in the analysis of variance. J. Am. Stat. Assoc..

[42] Wilcoxon F. (1945). Individual comparisons by ranking methods. Biom. Bull..

[43] Li X., Rytkin E., Zhao Q., Bhat P., Pfenniger A., Yin L., Huang X., Yang L., Yang B., Burrell A. (2025). High-resolution liquid metal–based stretchable electronics enabled by colloidal self-assembly and microtransfer printing. Sci. Adv..

